# Fecal Microbial and Metabolic Profiles in Dogs With Acute Diarrhea Receiving Either Fecal Microbiota Transplantation or Oral Metronidazole

**DOI:** 10.3389/fvets.2020.00192

**Published:** 2020-04-16

**Authors:** Jennifer Chaitman, Anna-Lena Ziese, Rachel Pilla, Yasushi Minamoto, Amanda B. Blake, Blake C. Guard, Anitha Isaiah, Jonathan A. Lidbury, Jörg M. Steiner, Stefan Unterer, Jan S. Suchodolski

**Affiliations:** ^1^Veterinary Internal Medicine and Allergy Specialists, New York, NY, United States; ^2^Clinic of Small Animal Medicine, Centre for Clinical Veterinary Medicine, LMU Munich, Munich, Germany; ^3^Gastrointestinal Laboratory, Department of Small Animal Clinical Sciences, Texas A&M University, College Station, TX, United States

**Keywords:** metronidazole, fecal micriobiota transplantation, dog, diarrhea, bile acids

## Abstract

The aim was to characterize differences in fecal consistency, and fecal microbiota and metabolome profiles in dogs with acute diarrhea (AD) treated with either fecal microbiota transplantation as enema (FMT; *n* = 11) or oral metronidazole (MET; *n* = 7) for 7 days. On days 0, 7, and 28 fecal samples were obtained. Fecal samples from healthy dogs (HC; *n* = 14) were used for comparison. Samples were analyzed by the previously validated qPCR based canine Dysbiosis Index (DI; increased values indicate microbiota dysbiosis) and 16S rRNA gene sequencing. The fecal metabolome was analyzed using a previously validated targeted canine assay for fecal unconjugated bile acids, and untargeted metabolomics. Fecal consistency improved significantly in dogs treated with FMT and MET by day 7 and day 28 (*p* < 0.01) compared to day 0. However, on day 28 fecal consistency was significantly better in FMT compared to MET (*p* = 0.040). At day 0, dogs with AD had an altered microbiota indicated by significantly increased DI, decreased alpha-diversity, and altered beta-diversity. In the FMT group, the DI decreased over time, while MET led to a significant increase in the dysbiosis index at day 7 and 28 compared to FMT. Sequencing data revealed that in FMT microbial diversity and beta-diversity was similar to HC at day 28, while in MET these parameters were still significantly different from HC. In dogs treated with FMT, a decrease in cholic acid and the percentage of primary bile acids was observed, whereas treatment with metronidazole led to an increase in cholic acid at day 7 and an increase in percentage of primary bile acids over time. Based on untargeted metabolomics, dogs with AD had an altered fecal metabolome compared to HC. Dogs treated with FMT clustered closer to HC at day 28, while dogs treated with MET did not. In this pilot study, dogs with AD had significant differences in fecal microbiota and metabolome profiles. Dogs treated with MET still had altered microbial and metabolic profiles at day 28 compared to dogs treated with FMT or healthy dogs.

## Introduction

Acute diarrhea (AD) is a common presenting complaint in dogs in everyday primary veterinary practice. Antibiotics are the most commonly used first line treatment for AD in the dog, regardless of the underlying cause. Surveys showed that 50–71% of dogs with AD presented to a veterinarian were treated with antimicrobials (1–3), although only a minority (3.2%) of these animals were tested for infectious agents (3). Only very recent studies have examined whether administration of antimicrobials does lead to shorter duration of diarrhea, and the results remain unclear. While one study has shown metronidazole to shorten duration of acute diarrhea (mean duration 2.1 vs. 3.6 days for placebo), another study did not reveal statistical differences in the duration of diarrhea when dogs were treated either with a probiotic (mean duration 3.5 days) metronidazole (4.6 days), or placebo (4.8 days) (4, 5).

Antibiotics have been associated with potential negative consequences. The intestinal tract is inhabited by ~100 trillion microbial cells that represent a complex ecosystem, the intestinal microbiota. The composition and adequate function of the microbiome play a crucial role in host health. Intestinal bacteria are important contributors to host metabolism. Studies have shown that antibiotics alter the gut microbiota, leading to lower microbial diversity, changes in specific bacterial taxa abundance and metabolic shifts (6–8). In humans and companion animals, antibiotic use is associated with development of diarrhea in a subset of patients (7, 9). Intestinal dysbiosis associated with antibiotic use, especially during early life stages, has also been associated with increased risk for inflammatory bowel disease (10), as well as atopy (11), or obesity (12) in humans.

Fecal microbiota transplantation (FMT) is the transfer of intestinal contents from a healthy donor to a diseased recipient with the aim to lessen diarrhea and improve gut health. In humans, FMT has been widely studied in humans with diarrhea and has high success rates in treating *Clostridium difficile* infections that are refractory to antibiotics (13). FMT has also been shown to restore fecal bile acid composition in association with recurrent *Clostridium difficile* infection (14). FMT has been reported in dogs with parvovirus infection (15), idiopathic inflammatory bowel disease (IBD) (16), and treatment of *Clostridium difficile*-associated diarrhea (17).

Most studies evaluating the impact of antibiotics on the intestinal microbiota have been performed in healthy animals, and little is known whether antibiotics lead to a normalization of the microbiota in dogs with intestinal disease, as is often used as the rationale for antibiotic use in acute diarrhea. Because of the above-mentioned potential negative consequences of antibiotic use on the intestinal microbiota, it is important to better characterize the microbial and metabolic changes that occur in AD, and how microbiota manipulation with two contrary microbiota treatments may affect these changes over time. These data may help design novel approaches to treatment of acute diarrhea.

Therefore, the aim of this pilot study was to evaluate differences in the microbiota and metabolome as well as clinical improvement in dogs with acute uncomplicated diarrhea after treatment with either fecal microbiota transplantation or oral antibiotic therapy with metronidazole. In this study we evaluated the fecal microbiota and metabolome using validated targeted qPCR assays for dysbiosis in dogs (18), and validated targeted assays for measuring fecal unconjugated bile acid concentrations in dogs (19, 20). Additionally we evaluated global shifts in the metabolome using untargeted fecal metabolomics, and in the microbiota using 16S rRNA gene sequencing.

## Materials and Methods

### Animals and Sampling

This study was a prospective treatment trial. The study protocol was approved by the Texas A&M University Institutional Animal Care and Use Committee (IACUC) (Protocol Number; AUP IACUC 2017-0094 CA). Owners were informed about the purpose of the study, and all owners signed a written informed consent form.

### Dogs With Acute Diarrhea

Between October 2016 and June 2017, 18 dogs were presented to Veterinary Internal Medicine and Allergy Specialists, New York City, United States with acute diarrhea (AD) lasting under 14 days. Day 0 was defined as the day of clinical presentation and study inclusion. A standardized history, physical examination, and fecal examination via flotation and PCR panel for enteropathogens (Diarrhea Panel, IDEXX Laboratories Inc., Westbrook, ME, USA) was performed in all patients. Inclusion criteria were acute onset of diarrhea with or without vomiting lasting <14 days. Exclusion criteria were severe dehydration (>7%), signs for systemic inflammation based on clinical examination (e.g., hyperthermia > 39.5°C, tachycardia, tachypnea, poor general condition), requirement for hospitalization, intestinal parasites, known history of chronic gastrointestinal signs, or pre-treatment with antibiotics or drugs known to cause mucosal irritation (e.g., non-steroidal anti-inflammatory drugs, corticosteroids) within 2 weeks of presentation. Fecal samples were collected by owners at the day of the appointment on days 0, 7, and 28, transported in a cooler box to the clinic. There the samples were frozen and stored at −20°C for up to 1 month before they were shipped for DNA extraction and analysis.

### Healthy Control Dogs

All healthy control dogs (HC) were privately owned and lived in diverse home environments in the same geographical area. These samples were collected as part of a larger study evaluating the fecal microbiota across different locations and were collected by owners and brought to the hospital for storage and shipping as described for diseased dogs above. All dogs were fed different commercial diets and none had a history of gastrointestinal signs or administration of antibiotics. Fecal samples from healthy control dogs were collected at a single time point during the same time period as the diseased dogs.

### Stool Donor

The stool donor for the fecal transplant was a privately owned, 3-year-old male neutered mixed breed dog living in New York City, USA, with no history of gastrointestinal disease, no history of antibiotic treatment, a normal body condition score and fecal score, and was fed various hypoallergenic diets during the study period (Natural Balance, Limited Ingredient Diets® Sweet Potato & Bison, Sweet Potato and Venison dry dog food, Purina ProPlan Veterinary Diets HA Vegetarian Formula, and Natural Balance Limited Ingredient Treats Sweet Potato and Bison Formula). The donor feces were screened for parasites, enteropathogens, and a normal microbiota using the dysbiosis index (18). Stool was collected on a regular basis (daily to every other day) into a plastic bag, which was labeled with the date, and frozen and stored at −20°C until processing for FMT of the day of each procedure.

### Treatment

Dogs with AD (*n* = 18) were divided into a fecal microbiota transplantation group (FMT, *n* = 11) and a metronidazole group (MET, *n* = 7) based on the owners' willingness to agree to one of the treatments. Ten dogs in the FMT group received a single FMT with 5 g of frozen donor stool per kg bodyweight (BW). The remaining dog, due to his size (27.5 kg) and availability of feces at day of transfer received 2.5 g of donor stool per kg BW. The donor feces was mixed with non-bacteriostatic 60 ml (small breed) to 120 ml (large breed) 0.9% NaCl on day 0 and given as a rectal enema. The frozen stool was mixed with the saline in a blender and blended for ~5 min. The solution was drawn up in a catheter tip 60 ml-syringe with a 12 French red rubber catheter attached. The fecal material was pushed into the catheter to avoid transplantation of air. To administer the enema, a small amount of non-bacteriostatic lubricant was put on the catheter, and the catheter completely introduced into the colon. To lessen the chances of a premature bowel movement, the recipient dog was not fed and the activity restricted for 4–6 h after the transplantation. Dogs in the MET group received 15 mg/kg metronidazole p.o. q12 h for 7 days. Additional treatment consisted of an antiemetic (maropitant at 1 mg/kg s.c. q24 h; Cerenia®, Zoetis) in case of vomiting. All patients did not receive any further treatment and the diet was not standardized.

### Evaluation of Clinical Signs

The clinical course in AD was evaluated by assessing the fecal consistency by a board-certified clinician using the Purina Fecal Scoring Chart on days 0, 7, and 28 in both groups. A lower fecal score indicates a more normal stool consistency.

### Analysis of Fecal Microbiota

#### Quantitative Real-Time PCR and Dysbiosis Index

To evaluate the fecal microbiota, fecal samples from the healthy control dogs at one single time point, and fecal samples from 18 dogs with acute diarrhea (11 treated with FMT, seven treated with metronidazole) from days 0, 7, and 28 were analyzed. A MoBio PowerSoil® DNA isolation kit (MoBio Laboratories, Carlsbad, CA, USA) was used according to the manufacturer's instructions for DNA extraction of 100 mg each from each fecal sample. Quantitative PCR assays (qPCR) were performed for key bacterial taxa known to be altered in dogs with gastrointestinal disease (i.e., total bacteria, *Faecalibacterium, Turicibacter, Escherichia coli, Streptococcus, Blautia, Fusobacterium*, and *Clostridium hiranonis*) as previously described (18). PCR conditions were as follows: 2 min at 95°C, 40 cycles for 5 s at 95°C, and 10 s at the optimized annealing temperature with 10 μL of SYBR-based reaction mixtures [5 μL of SsoFast™ EvaGreen® supermix (Biorad Laboratories)], 1.6 μL of high quality PCR water, 0.4 μL of each primer (final concentration: 400 nM), 1 μL of 1% BSA (final concentration: 0.1%), and 2 μL of DNA (1:10 or 1:100 dilution). The qPCR results were expressed as the log amount of DNA (fg) for each bacterial taxa/10 ng of isolated total DNA. The results of the qPCR assays were also combined to calculate the Dysbiosis Index, which expresses the degree of dysbiosis as a single numeric value. A negative DI indicates normobiosis, whereas a positive DI indicates dysbiosis (18).

#### 16S rRNA Gene Sequencing

The V4 region of the 16S rRNA gene was sequenced at MR DNA (www.mrdnalab.com, Shallowater, TX, USA). Briefly, PCR primers 515F/806R were used with the HotStarTaq Plus Master Mix Kit (Qiagen, USA) in a 28-cycle PCR for sample amplification under the following conditions: 3 min at 94°C, 28 cycles of 30 s at 94°C, 40 s at 53°C, 1 min at 72°C, and final elongation for 5 min at 72°C. A DNA library was set up using the Illumina TruSeq DNA library preparation protocol. Illumina MiSeq was used for sequencing following the manufacturer's guidelines, and sequence data were uploaded into the NCBI GenBank database under submission number SRP174116.

#### Analysis of Sequences

For processing and analysis of sequences, QIIME v 1.9 (Quantitative Insights Into Microbial Ecology) was used as previously described (21). Using default settings, raw sequence data were demultiplexed, and low quality results were filtered. USEARCH was used to detect and delete chimeric sequences (22). Remaining sequences were assigned to operational taxonomic units (OTUs) with an open-reference picking protocol in QIIME against the 97% clustered sequences from Greengenes database (23). Alpha diversity was assessed with Chao 1, Shannon diversity, and observed species. Beta diversity, based on weighted UniFrac distances, was visualized with Principal Coordinate Analysis (PCoA) plots.

### Analysis of the Fecal Metabolome

#### Targeted Metabolomics Approach for Measurement of Fecal Unconjugated Bile Acids

Fecal samples from 14 healthy control dogs from a single time point, and fecal samples from 18 dogs with acute diarrhea (11 treated with FMT and 7 treated with metronidazole) from days 0, 7, and 28 were used for measurement of unconjugated bile acids (BA) concentrations with previously described methods (19, 20). Feces were lyophilized and 10–15 mg of the lyophilized material were used for downstream extraction. Two hundred μL of butanol (with internal standards CA-d4 and LCA-d4) and 20 μL of 37% HCl were added to each fecal sample. The samples were then vortexed for 30 s and incubated for 4 h at 65°C. Under nitrogen gas, samples were evaporated until dryness at 65°C for ~25 min. Twohundred μL of derivatization agent (HMDS+TMCS+Pyridine,3:1:9, Sigma-Aldrich, St. Louis, Missouri) were added to each sample and samples were incubated at 65°C for 30 min followed by a second evaporation under nitrogen gas until dryness at 65°C (~25 min). Afterwards, samples were resuspended in 200 μL of hexane, vortexed briefly, and centrifuged at 4°C for 10 min at 3,000 rcf. For further downstream analysis, an 80 μL aliquot was transferred to a gas chromatography-mass spectrometry (GC/MS) vial. As described previously, a GC/MS system (6890N and 5975 inert Mass Selective Detector, Agilent, Santa Clara, California) was used (19, 20). Cholic acid, chenodeoxycholic acid, lithocholic acid, deoxycholic acid, and ursodeoxycholic acid were measured. To normalize for variable starting fecal weights, concentrations of BAs were calculated according to the aliquot weight and expressed in μg/mg of lyophilized fecal content. Additionally, they were expressed as percent of total BAs measured, as well as total primary BA (sum of cholic acid and chenodeoxycholic acid) and total secondary BA (sum of lithocholic acid, deoxycholic acid, and ursodeoxycholic acid).

#### Untargeted Metabolomics Approach

To evaluate the fecal metabolome, fecal samples from 14 healthy dogs from a single time point, and fecal samples from 17 dogs with acute diarrhea (10 treated with FMT, seven treated with metronidazole) from days 0, 7, and 28 were used. In one patient of the FMT group, untargeted analysis of fecal metabolome could not be performed due to low sample volume available.

Fecal samples were analyzed at the West Coast Metabolomics Core Facility (University of California, Davis, CA, USA) by using gas chromatography time-of-flight mass spectrometry (GC-TOF-MS). Briefly, 10 mg of each sample were homogenized, extracted, and centrifuged. The supernatant was dried and retention index marking fatty acid methyl esters were added, and a derivatization step using methoxyamine hydrochloride and N-methyl-N-trimethylsilyl trifluoroacetamide with 1% trimethylchlorosilane was performed. A volume of 0.5 μL was injected onto a Restek rtx5Sil- MS column in splitless mode on a temperature-gradient programmed gas chromatograph (oven 50°C to 330°C at 20°C/min, injector 50°C to 250°C at 12 C/sec) coupled with Leco Pegasus IV time-of-flight mass spectrometer (scanning 17 spectra/sec from 80 to 500 Da, −70 eV ionization energy, 1,800 V detector voltage) with helium carrier gas (1 mL/min), For raw data processing ChromaTOF v. 2.32. software was used. Analysis of metabolites was performed as previously described (24, 25). Metabolites of unknown identity were deleted from the data table and then peak heights were uploaded to MetaboAnalyst 4.0 (https://www.metaboanalyst.ca/), followed by log transformation and Pareto scaling for normalization of the data.

Due to equipment difficulties during the untargeted metabolomics analysis, samples from the MET group and FMT group needed to be analyzed in separate runs for untargeted metabolomics. However, the HC group was re-analyzed in each of these runs together with FMT and MET. Therefore, it was appropriate to statistically compare the HC group vs. FMT, and HC group vs. MET, but due to potential batch effects, we did not compare directly FMT vs. MET over time for the untargeted metabolomics data.

### Statistical Analysis

All datasets were tested for normal distribution using D'Agostino & Pearson omnibus normality tests. Dog characteristics (i.e., age, breed, gender, and body weight) from FMT, MET, and HC were compared using a Kruskal-Wallis test.

The qPCR data and targeted fecal bile acid metabolites were statistically analyzed using 2-way repeated measures ANOVA, with treatment (FMT vs. MET) and time points as class variables, followed by a Tukey's multiple comparison test.

To evaluate differences in the microbiota (i.e., alpha diversity and relative abundance in 16S rRNA sequencing and qPCR data) and metabolites between healthy dogs and dogs with acute diarrhea in each group, unpaired *t*-tests or Mann-Whitney tests were used as appropriate. For assessment of microbial and metabolic changes as well as for changes in fecal scores within groups over time, data from days 0, 7, and 28 were compared using a Friedman test and Dunn's multiple comparison test. Additionally, time points from FMT and MET were also compared to HC using an unpaired *t*-test or Mann-Whitney test as appropriate. All *p*-values were adjusted for multiple comparisons using Benjamini and Hochberg False discovery rate. Significance was set at p<0.05. All statistics were performed with GraphPad Prism 8 (GraphPad Software Inc., San Diego, USA).

## Results

### Animals

Patient characteristics are summarized in Table 1. There was no significant difference in age (*p* = 0.839) or body weight (*p* = 0.360) between FMT, MET, and HC. Groups showed a significant difference in gender (*p* = 0.031).

**Table 1:** Patient characteristics.

	**HC**	**FMT**	**MET**	***p*-value**
Age in years median (range)	4.5 (1.0–12.0)	4.0 (0.5–13)	7.0 (2.0–9.0)	*p* = 0.839
Weight in kg median (range)	21.8 (2.3–49.9)	12.9 (2.5–30.0)	7.0 (4.3–39.1)	*p* = 0.360
Gender female/male	6/7	8/2	1/6	*p* = 0.031

*HC, healthy controls; FMT, fecal transplant group; MET, metronidazole group*.

### Fecal Scores

**Figure 1:**
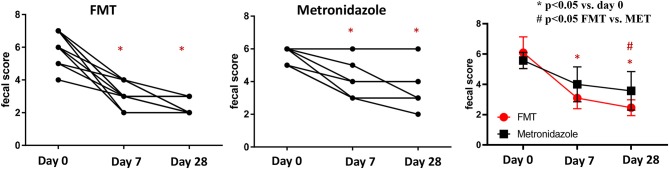
Fecal scores in dogs with acute diarrhea treated with either FMT as a single enema, or with metronidazole for 7 days (MET). A lower fecal score indicates a more normal stool consistency. In both groups a significant decrease in fecal scores was observed on day 7 (*p* < 0.001 in FMT and *p* = 0.017 in MET) compared to day 0. Fecal scores were significantly lower in the FMT compared to the MET group on day 28 (*p* = 0.040). FMT, fecal microbial transplantation; *p < 0.05 compared to day 0; # significant difference between FMT and MET at specific time points.

On day 0, the median fecal score for dogs in the FMT group was 6 (range 4–7) and that in the MET group also was 6 (5–6), which was not significantly different (*p* = 0.212; Figure 1). Fecal scores decreased significantly in both the FMT and MET groups at day 7 and day 28 when compared to day 0 (*p* < 0.001), respectively. However, fecal scores were significantly lower in the FMT group on day 28 compared to those in the MET group (*p* = 0.036; Figure 1).

### Microbial Analyses

#### Fecal Dysbiosis Index

**Figure 2:**
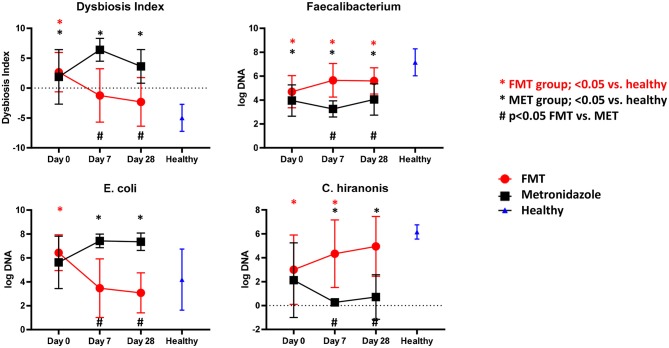
Results of qPCR. Fecal dysbiosis index (DI), and abundances of *Faecalibacterium, E. coli* and *C. hiranonis* in both treatment groups over time and relative to healthy dogs. Dogs after FMT showed a significant decrease in the DI and *E. coli*, which were not significantly different at days 7 and 28 compared to healthy dogs. In contrast, in dogs in the MET group showed a significant increase in the DI and abundance of *E. coli*, which were significantly increased at days 7 and 28 compared to healthy dogs. The abundance of *Faecalibacterium*, a bacterial taxon associated with health, increased numerically in the FMT group and was significantly higher compared to dogs in the MET group at days 7 and 28. However, the abundances remained still significantly lower in both groups when compared to healthy dogs. *C. hiranonis*, a bacterium important for conversion of primary to secondary bile acids in dogs, increased in abundance in the FMT group, which was no longer significantly different from healthy dogs at day 28. In contrast, dogs in the MET group showed a decrease in the abundance of *C. hiranonis* at day 7 (end of MET treatment) and day 28 when compared to healthy dogs.

There were no significant differences in any of the bacterial taxa and the DI between both treatment groups at day 0. Dogs with AD (Figure 2) had a significantly (*p* < 0.001) higher DI on day 0 with a median DI of 4.1 (range −4.5–6.5) compared to healthy control dogs (median DI of −4.6, range −8.4 to −0.6). The abundances of *Faecalibacterium* (*p* < 0.001), *Fusobacterium* (*p* = 0.004), *Blautia* (*p* = 0.010), *C. hiranonis* (*p* = 0.002), and *Turicibacter* (*p* = 0.004) were significantly lower in AD at day 0, while the abundances of *Streptococcus* spp. (*p* = 0.012) and *E. coli* (*p* = 0.046) were significantly greater compared to healthy dogs.

Dogs after FMT showed a significant decrease in the DI and *E. coli*, which were not significantly different at days 0 and 28 compared to healthy dogs. Dogs in the MET group showed a significant increase in the DI and abundance of *E. coli*, which were significantly greater at days 0 and 28 compared to healthy dogs.

The abundance of *Faecalibacterium*, a bacterial taxon associated with health, increased numerically in the FMT group and was significantly higher compared to dogs in the MET group at day 7 and 28. However, the abundances remained still significantly lower in both groups when compared to healthy dogs.

*C. hiranonis*, a bacterium important for conversion of primary to secondary bile acids in dogs, increased in abundance in the FMT group, which was not significantly different from healthy dogs at day 28. Dogs in the MET group showed a decrease in the abundance of *C. hiranonis* at day 7 (end of MET treatment) and day 28 when compared to healthy dogs.

#### 16S rRNA Gene Sequencing—Alpha Diversity Measures

**Figure 3:**
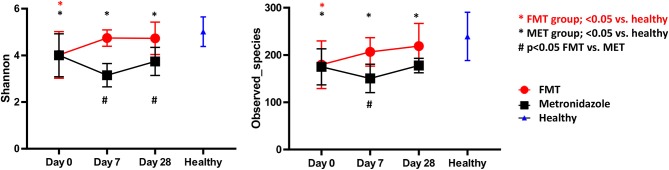
Shannon Index (left panel) and species richness (right panel) for healthy control dogs vs. dogs with acute diarrhea treated with either FMT as a single enema, or with metronidazole (MET) for 7 days. Both Shannon and species richness were significantly lower at day 0 vs. healthy dogs. Dogs in the FMT group showed a significant increase in both parameters, which were no longer significantly different at days 7 and 28 compared to healthy dogs. In contrast, dogs in the MET group remained significantly lower in both parameters at days 7 and 28 when compared to healthy dogs.

There were no significant differences in any of the alpha diversity measures at day 0 between the two treatment groups. Statistical analysis (S1 Table; Figure 3) revealed that dogs with acute diarrhea in both groups had significantly reduced alpha diversity (observed_species, Shannon index) compared to healthy dogs at day 0. Dogs in the FMT group showed an increase in alpha diversity measures (S1 Table; Figure 3), which were not significantly different at days 7 and 28 compared to healthy dogs. In contrast, dogs in the MET group showed a significant decrease in both parameters at day 7 and 28 when compared to healthy dogs.

#### 16S rRNA Gene Sequencing—Changes in Microbial Communities (Beta-Diversity)

There were no significant differences between the FMT and MET group at day 0 based on weighted unifrac distances (Analysis of similarities [ANOSIM], *R* = 0.137, *p* = 0.943).

**Figure 4:**
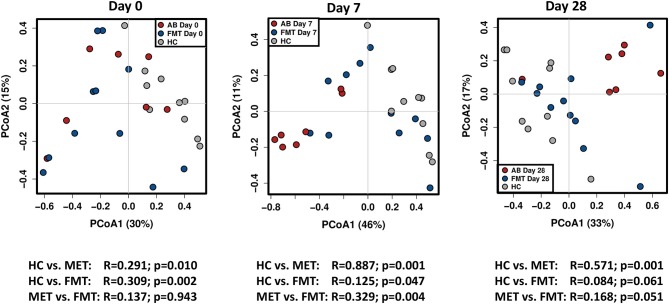
PCoA plots based on weighted unifrac distances for healthy control dogs (HC) vs. dogs with acute diarrhea treated with either FMT as a single enema, or with metronidazole (MET). Statistics are based on ANOSIM (R-value indicates size effect). There were no significant differences between the FMT and MET group at day 0, but both groups were significantly different from HC. After FMT, microbial communities clustered closer to healthy dogs at day 7, and were not significantly different from HC at day 28. In contrast, administration of MET showed a larger difference in microbial communities compared to healthy dogs at day 7 (based on an increased effect size *R* = 0.887), and microbial communities were still significantly different compared to HC at day 28.

Both groups were significantly different from HC (Figure 4). For the FMT group vs. HC, statistical analysis was as following: weighted unifrac distances ANOSIM *R* = 0.309, *p* = 0.002. For the MET group vs. HC: weighted unifrac distances ANOSIM *R* = 0.291, *p* = 0.010.

On day 7 and day 28 after FMT, microbial communities showed a trend to cluster closer to the healthy controls (based on decreasing ANOSIM effect size), and were not significantly different from healthy controls ay day 28 based on weighted distances (ANOSIM *R* = 0.084, *p* = 0.061).

In contrast, based on the ANOSIM effect size, the microbiota in the MET group showed a larger difference compared to HC at day 7 (end of MET administration). At day 28 (Figure 4), microbial communities were still significantly different from healthy dogs based weighted distances (ANOSIM *R* = 0.571, *p* = 0.001). Results for unweighted unifrac distances were similar and are shown as Supplemental Figure 1.

#### Bacterial Taxa

Univariate statistics showed several significant differences in bacterial taxa between dogs with acute diarrhea in both groups and healthy controls at day 0 (S1 Table). At the phylum level, dogs with AD had a significantly lower abundance of Bacteroidetes (*p* = 0.003) and Fusobacteria (*p* = 0.029). At the family level, dogs with AD had significantly higher abundances of Enterobacteriaceae (*p* = 0.006), Corynebacteriaceae (*p* = 0.011), Bifidobacteriaceae (*p* = 0.001), Staphylococcaceae (*p* = 0.008), Lactobacillaceae (*p* = 0.020), and Leuconostocaceae (*p* = 0.001). The abundances of Bacteroidaceae (*p* = 0.006), Fusobacteriaceae (*p* = 0.029), Ruminococcaceae (*p* = 0.024), Veillonellaceae (*p* < 0.001), and Alcaligenaceae (*p* = 0.005) were significantly decreased in dogs with AD compared to HC.

#### Fecal Bile Acid Concentrations

**Figure 5:**
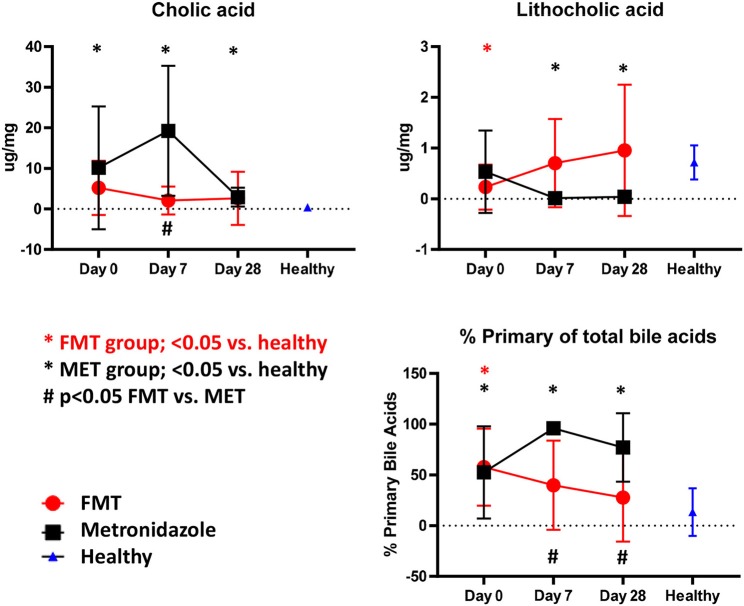
Fecal unconjugated bile acid concentrations in both treatment groups and relative to healthy dogs. Dogs in the FMT group showed a normalization of bile acid profiles, which were not significantly different from healthy dogs at days 7 and 28 for cholic acid, lithocholic acid, and the percentage of primary bile acids. In contrast, dogs in the MET group showed significantly abnormal concentrations of these parameters when compared to healthy dogs at days 7 and 28.

Compared to healthy control dogs (Figure 5), the FMT (*p* = 0.054) and MET group (*p* = 0.012) at day 0 had higher fecal concentration of the primary bile acid colic acid, and increased percentage of primary bile acids as part of the total analyzed fecal bile acid pool (FMT group, *p* = 0.007; MET group, *p* = 0.357). Of the secondary bile acids, lithocholic acid was significantly lower (*p* = 0.0016), while deoxycholic acid was numerically lower (*p* = 0.080). Fecal microbiota transplantation (FMT) led to a significant decrease in the percentage of primary bile acids at day 28 compared to day 0, whereas MET led to a significant increase at day 7. The percentage of primary bile acids was also lower in the FMT group at day 7. For day 7 and 28 the percentage of primary bile acids in the FMT group approached values in heathy dogs (Figure 5). Fecal concentrations of cholic acid were also significantly higher in the MET group at day 7 compared to FMT. Trends for an increase were observed for the secondary bile acid lithocholic acid after FMT but this did not reach significance (*p* = 0.189). There was a significant negative correlation between the abundance (based on quantitative PCR) of the 7α-dehydroxylating bacterium *C. hiranonis* and fecal concentrations of primary bile acids (Spearman r = −0.575; *p* < 0.0001) and the percentage of fecal primary bile acids (Spearman r = −0.651; *p* < 0.0001).

### Untargeted Fecal Metabolomics

**Figure 6:**
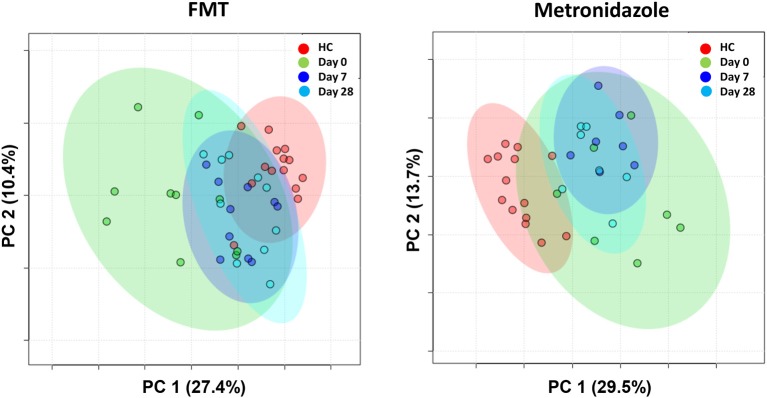
PCA plots showing changes based on untargeted metabolomics in healthy control dogs (HC) vs. dogs with acute diarrhea treated with either FMT as a single enema, or with metronidazole. Dogs with acute diarrhea treated with fecal microbiota transplantation (FMT) clustered closer to healthy dogs on day 28. Dogs with acute diarrhea treated with metronidazole did not cluster closer to healthy dogs on day 28 than before.

**Figure 7:**
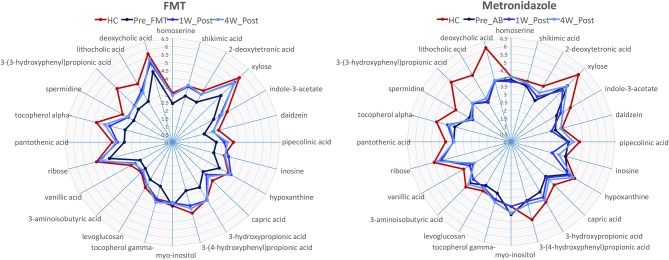
Spider plots showing changes in selected metabolites in healthy control dogs (HC) vs. dogs with acute diarrhea treated with either FMT or with metronidazole. The spider plots illustrate visually how the most significantly different metabolites based on univariate analysis between HC and treatment groups (S2 Table) changed after treatment in each group. The median abundance of metabolites in the FMT group approximated the abundance of metabolites in healthy dogs on days 7 and 28 after FMT. In contrast, most metabolites which were altered in the MET at day 0, did not change in abundance after treatment with metronidazole (Numbers on axis indicate the log median value for each compound).

A total of 237 and 261 metabolites were measured in the FMT and MET group, respectively. Of those, 131 metabolites were significantly different from HC based on adjusted *p*-values in the FMT group, and 117 in the MET group. Therefore, plots based on Principal Component Analysis (PCA; Figure 6) revealed clear clustering of samples from day 0 compared to HC in both groups. Dogs with acute diarrhea treated with FMT clustered closer to healthy dogs on day 28. In contrast, dogs with acute diarrhea treated with metronidazole did not cluster closer to healthy dogs on day 28. Spider plots (Figure 7) illustrate visually how the most significantly different metabolites based on univariate analysis between HC and treatment groups (S2 Table) changed after treatment in each group. Generally, the median abundance of metabolites in the FMT group approximate the abundance of metabolites in healthy dogs on days 7 and 28 after FMT. In contrast, most metabolites which were altered in the MET at day 0, did not change in abundance after treatment with metronidazole.

## Discussion

Dogs with acute uncomplicated diarrhea showed significant alterations in the fecal microbiota (i.e., reduced microbial diversity, altered microbial communities, and an increased dysbiosis index), and significant alterations in the fecal metabolome compared to healthy dogs. These results are consistent with previous studies evaluating the microbiota in dogs with acute diarrhea. A study in 12 dogs with acute diarrhea revealed similar changes as observed in this study with significant decreases in *Faecalibacterium, Turicibacter*, and Bacteroidetes, and increases in *C. perfringens* (26). Similar changes in the fecal microbiota were also reported in another study evaluating 13 dogs with acute diarrhea, with Bacteroidetes, *Faecalibacterium*, and an unclassified *Ruminococcus* genus being underrepresented, while the abundance of the genus *Clostridium* was increased (27). The qPCR based dysbiosis index was developed in dogs with chronic enteropathies (18), however, there are common findings of altered bacterial communities between dogs with acute and chronic diarrhea (26–28). In this study, several bacterial taxa that are typically altered in chronic enteropathies were also altered in dogs with acute diarrhea. These included decreases in *Faecalibacterium, Fusobacteria, Blautia, C. hiranonis*, and *Turicibacter*, with several of these changes having also been reported in previous studies (26–28). Therefore, dogs with acute diarrhea showed a significant increase in the qPCR based dysbiosis index.

One pathway that is now understood as being important in the pathophysiology of diarrhea in humans is the intestinal bile acid pathway (29–31). *C. difficile* infections are associated with bile acid dysmetabolism, with increased primary and decreased secondary bile acids (14). The intestinal bile acid pathway has not yet been evaluated in dogs with acute diarrhea. With a validated assay for canine fecal samples (19) we confirmed that dogs with AD had higher fecal concentrations of the primary bile acid cholic acid, and a significantly higher percentage of primary bile acids compared to healthy controls. A recent study reported that dogs with chronic inflammatory enteropathy (CE) have a decreased expression of apical sodium-dependent bile acid transporter, potentially leading to malabsorption of bile acids in the ileum and an increased percentage of primary bile acids in feces (20). Furthermore, the fecal dysbiosis and especially a decrease in the abundance of 7α-dehydroxylating bacterium *C. hiranonis* correlated significantly with a decreased percentage of secondary bile acids in dogs with CE (20). It is likely that the decrease of *C. hiranonis* observed in the present study in dogs with acute diarrhea, led to insufficient conversion of primary to secondary bile acids (32), and, therefore, was at least partially responsible for the increase in percentage of primary bile acids. FMT treatment led to decreases in the percentage of primary bile acids over time when compared to healthy control dogs. In contrast, treatment with metronidazole did not lead to normalization of these bile acid profiles. The decrease in primary bile acids after FMT but not after antibiotic treatment is consistent with data in humans with *C. difficile* infection receiving FMT (14). FMT may restore fecal bile acid composition by replenishing colonic bacteria, which deconjugate and dehydroxylate the primary bile acids cholic and chenodeoxycholic acid to form the secondary bile acids deoxycholic and lithocholic acid. However, metronidazole may target intestinal bacteria such as *C. hiranonis* or *C. scindens*, which convert primary bile acids to secondary bile acids, resulting in decreased production of lithocholic acid and deoxycholic acid. This is evident in this study, as *C. hiranonis* turned abnormally low after treatment with metronidazole (Figure 2), but increased after FMT, and there was significant correlation with the abundance of *C. hiranonis* and primary bile acids. Increased primary bile acids can be a cause of diarrhea, but it is unclear at what concentrations this may occur and how it relates to interactions between other metabolites and bacteria (31, 33). It is currently unknown why fecal scores improved in the MET group, even when primary bile acids remained increased.

Untargeted metabolomics is a relatively new field and there are only few studies reported, especially for fecal samples. In human patients with idiopathic inflammatory bowel disease (IBD), an untargeted metabolomics approach on stool samples revealed several alterations in metabolic pathways (34). An untargeted profiling of serum metabolites in dogs with IBD showed increased abundances of 3-hydroxybutyrate, hexuronic acid, ribose, and gluconic acid lactone compared to healthy dogs, suggesting the presence of oxidative stress (25). Fecal metabolomics in healthy dogs fed bones and raw food diet showed increased abundances of 4-hydroxybutyric acid and 4-aminobutyric acid when compared to dogs fed commercial diets (35). One study reported untargeted metabolomic analysis of serum and targeted analysis for fecal short-chain-fatty acids (SCFA) in dogs with acute diarrhea. In that study, dogs with acute diarrhea showed alterations in the tryptophan-serotonin-indole pathway in serum, and decreased fecal propionic acid compared to healthy dogs (27).

In this current study, we used an untargeted metabolomics approach to evaluate whether fecal metabolites in dogs with acute diarrhea are altered compared to healthy control dogs, and to study the effect to two treatment approaches. The most significant differences between dogs with acute diarrhea and healthy control dogs were linked to several metabolic pathways, including amino acid metabolism (i.e., homoserine and pipecolonic acid), cholesterol and lipid metabolism (i.e., zymosterol, dihydrocholesterol, and beta-sitosterol), and tryptophan metabolism (i.e., indole-3-lactate and nicotinic acid).

In a recent study, parvovirus-infected puppies that received FMT in addition to standard therapy with antibiotics showed a faster resolution of diarrhea compared to dogs that only received standard therapy with antibiotics, suggesting a benefit of FMT on fecal diarrhea scores (15). While dogs in both the FMT and the MET group showed improved fecal consistency over time, fecal scores were significantly lower in dogs that received FMT compared to the MET group on day 28. The differences in fecal scores based on treatment observed in the current pilot study could also be due to the small sample size in both groups and especially the metronidazole group, and larger sized groups are needed to evaluate whether FMT can lead to faster resolution of diarrhea compared to antibiotic treatment. However, the current study showed clear differences on the effect of FMT vs. metronidazole on the fecal microbiota and metabolome over time. Dogs that received FMT showed normalization of the microbiota as indicated by an increase in microbial diversity, a microbiota profile that clustered closer to healthy control dogs, and also a significant decrease in the dysbiosis index. There are no comprehensive studies that evaluated changes in the fecal microbiota in dogs with intestinal disease after FMT. However, FMT trials in humans with *C. difficile* infection also typically show an increase in microbial diversity and changes toward microbial profiles of healthy controls (36, 37).

In contrast, the microbiota of dogs receiving MET showed persistent dysbiosis even at 28 days, as indicated by a significant increase in the dysbiosis index and increased R-values in unifrac distances. Studies in healthy dogs have shown that commonly used antibiotics such as tylosin (6, 38) or metronidazole (39) have a profound and lasting effect on microbial composition in the small and large intestine. Notably, metronidazole administered to healthy dogs led to decreased fecal abundances in Fusobacteriaceae and Turicibacteraceae, and increases in Enterobacteriaceae and Streptococcaceae. In the current study dogs with acute diarrhea showed changes in the fecal microbiota after the administration of metronidazole similar to changes in healthy dogs receiving metronidazole as described in the previous study (39). Since the mentioned bacterial groups are also part of the dysbiosis index (18), this explains why the index increased after antibiotic therapy. Little is known about the effect of antibiotics on the microbiota in dogs with intestinal disease. Commonly used antibiotics like metronidazole or tylosin may have immunomodulatory effects, but they may also work by reducing total bacterial load in the small intestine, for example in dogs with bacterial overgrowth due to exocrine pancreatic insufficiency, which was reported to correlate with improvement of diarrhea (40). A reduction in total bacterial load may also be a mechanism that may lead to improvement in fecal scores over time in dogs treated with metronidazole. However, the current study clearly suggests that metronidazole has negative and long-lasting effects on the fecal microbiota even after cessation of administration, and this needs to be considered in the treatment decision for acute diarrhea.

Similarly, clear differences were observed in the fecal metabolome between the two treatment strategies. Measuring fecal metabolites allows assessment of functional changes in the intestinal microbiota, but also assessment of abnormalities in host-derived metabolites (24, 41). Therefore, bacterial-derived metabolites would be expected to correlate with improvement or worsening of microbial dysbiosis. Consequently, the PCA plots (Figure 6) showed that the fecal metabolome in the FMT group after 28 days clustered closer to healthy controls. In contrast, the fecal metabolome remained largely unchanged after treatment with metronidazole and clustered away from the healthy controls even after 28 days. The spider plots depicted in Figure 7 illustrate that several fecal metabolites that were abnormal on day 0 in dogs with acute diarrhea, approximated the mean abundance found in healthy dogs on day 28 after FMT. In contrast, after treatment with metronidazole, most of those same metabolites did not approximate the mean abundances found in healthy dogs. This data suggest that FMT helps to restore the fecal metabolome, whereas metronidazole does not. Because some metabolites remain altered after metronidazole treatment, these findings would suggest that the impact of AD is mostly on bacterially-derived metabolites, rather than host-metabolites. Since the metabolomic field is relatively new, limited data is available about the impact of different treatment regimens on the fecal metabolome, especially in patients with acute diarrhea. In children with pediatric ulcerative colitis, FMT led to normalization of some metabolites, which correlated with clinical improvement (42). Since acute diarrhea is likely not associated with major damage to the intestinal tract, it would be expected that most metabolomic changes are due to bacterial dysbiosis, and should revert after correction of dysbiosis. Of note, some metabolites, such as 3-(3-hydroxyphenyl)propionic acid, did not normalize when compared to healthy dogs with either treatment. Currently, the functions of many of these metabolites are unknown, and it can only be speculated why the abundance of these metabolites remained altered. For example, 3-(3-hydroxyphenyl)propionic acid is a metabolite of propionic acid, which has been shown to be decreased in dogs with acute diarrhea (27), and these data may show that even after an episode of acute diarrhea, some microbial functions remain altered for a prolonged period of time. Whether these changes need more time to resolve would need to be determined in studies with a longer follow-up interval.

While the microbiota and metabolome clustered closer to healthy dogs 28 days after receiving FMT, there were still alterations from the healthy control group. It is conceivable that the microbiota does not fully recover after an episode of acute diarrhea. Also, some of the altered metabolites may be host-derived and may indicate prolonged damage to host tissues. Future studies are needed to determine whether patients with AD resemble HC dogs after longer time periods of follow-up.

Limitations of this pilot study were the small sample size, lack of randomization, and lack of a placebo group, and that the evaluation of fecal scores was not performed by a blinded investigator and not performed daily in the first week. Patients were not randomized in this study, as FMT is a novel treatment option in companion animals, and pilot data needs to be generated to increase owner acceptance before larger scale studies can be initiated. For the same reason no placebo group was included. However, because at baseline there were no significant differences between treatment groups for all analytically measured parameters, lack of randomization is unlikely do have caused any bias in our results. We compared FMT to metronidazole in this study because, while acute uncomplicated diarrhea is often self-limiting, treatment with antibiotics is still initiated in 50% to 71% of patients in practice (2, 3). Just recent data would suggest that there is limited clinical benefit of giving metronidazole over placebo as both treatments had similar outcomes in clinical signs (4, 5), but both studies did not report effects on the microbiota and metabolome (4, 5). Therefore, future studies should also include comparisons between FMT and placebo to evaluate whether FMT treatment has improved outcomes in the metabolome and microbiota. Future consensus concerning best practices for sample collection and storage for metabolomic studies will also strengthen future studies. A study in humans with recurrent or refractory *C. difficile* infection did not find differences in resolution of diarrhea when FMT was performed with either fresh or frozen donor stool (43). However, it remains to be determined whether FMT in dogs with AD should be performed with fresh or frozen feces for optimal microbial and metabolic as well as clinical improvement. The optimal dose of donor feces remains also to be determined. In this study 10/11 dogs received a dose of 5 g donor feces per kg BW. The remaining dog received 2.5 grams/kg BW because of his weight (27.5 kg) and availability of donor feces at day of FMT. Despite receiving only half the dose, the fecal scores (7 at day 0, 4 at day 7, and 2 at day 28) and dysbiosis index (+4.5 at day 0, −1.0 at day 28, and −5.0 at day 28) were similar in response as for the other dogs. Finally, it would also be of interest to compare the donor microbiota to the recipient's microbiota after FMT in future studies.

In conclusion, in this pilot study, dogs with AD had significant differences in fecal microbiota and metabolome profiles. We observed that dogs with AD have abnormal fecal bile acid profiles. We observed differences in the response of the microbiota and metabolome to treatment with either FMT or metronidazole. Despite a similar improvement in fecal scores, dogs treated with MET still had altered microbial and metabolic profiles at day 28 compared to dogs treated with FMT or healthy dogs. This would suggest that use of metronidazole has a negative impact on the fecal microbiota in dogs with acute diarrhea, similar as previously reported in healthy dogs. Further studies are warranted to further explore these altered metabolic pathways and evaluate novel treatment approaches for acute diarrhea.

## Data Availability Statement

The datasets generated for this study can be found in the NCBI GenBank database under submission number SRP174116.

## Ethics Statement

The study protocol was approved by the Texas A&M University Institutional Animal Care and Use Committee (IACUC) (Protocol Number; AUP IACUC 2017-0094 CA). Owners were informed about the purpose of the study, and all owners signed a written informed consent form.

## Author Contributions

JC, A-LZ, SU, and JSS designed study. JC, RP, YM, AB, BG, and AI performed analysis. A-LZ, RP, YM, AI, JL, JMS, and JSS performed statistical analysis. All authors contributed to writing and editing the manuscript.

### Conflict of Interest

The authors declare that the research was conducted in the absence of any commercial or financial relationships that could be construed as a potential conflict of interest.
