# Supplementary material for: Fecal Microbial and Metabolic Profiles in Dogs With Acute Diarrhea Receiving Either Fecal Microbiota Transplantation or Oral Metronidazole
Source: Front Vet Sci. 2020 Apr 16;7:192. doi: 10.3389/fvets.2020.00192 (PMC7182012; doi:10.3389/fvets.2020.00192)
Supplement: Supplementary file 3 [file Image_1.pdf]

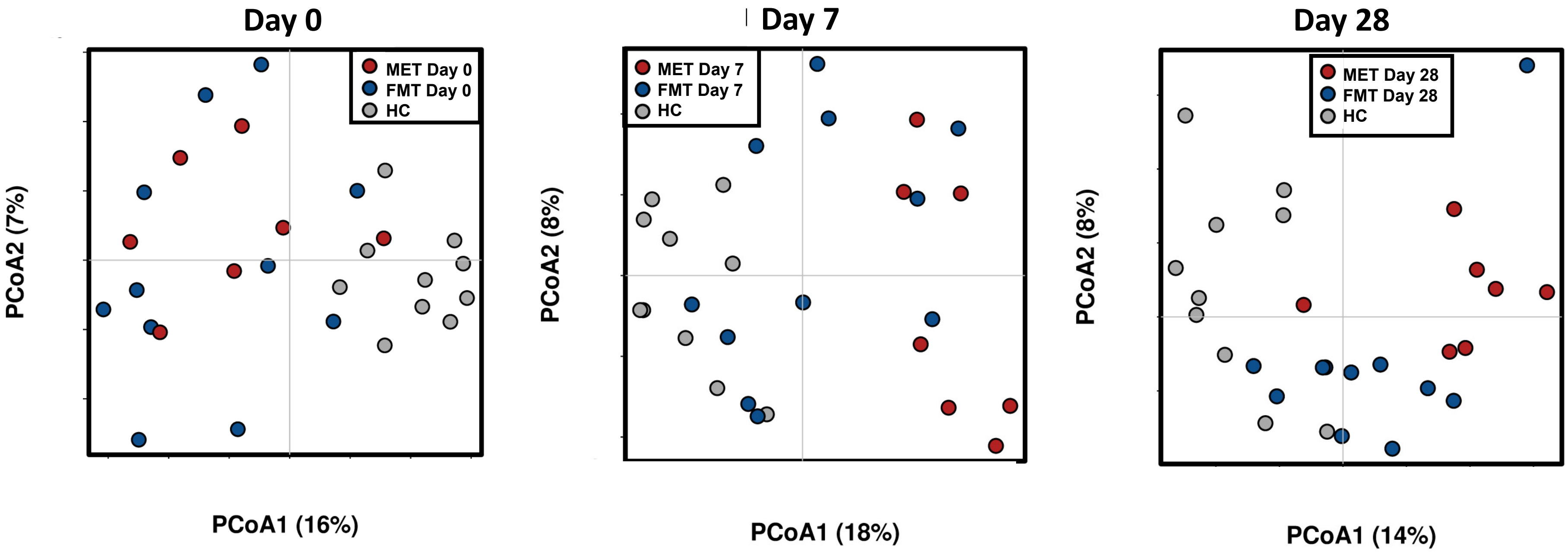

Day 0	Day 7	Day 28
HC vs. MET: R=0.571; p=0.001	HC vs. MET: R=0.946; p=0.001	HC vs. MET: R=0.772; p=0.001
HC vs. FMT: R=0.502; p=0.001	HC vs. FMT: R=0.246; p=0.003	HC vs. FMT: R=0.265; p=0.002
MET vs. FMT: R=0.148; p=0.954	MET vs. FMT: R=0.235; p=0.024	MET vs. FMT: R=0.306; p=0.004

Supplemental Fig 1. PCoA plots based on unweighted unifrac distances for healthy control dogs (HC) vs. dogs with acute diarrhea treated with either FMT as a single enema, or with metronidazole (MET). Statistics are based on ANOSIM (R-value indicates size effect). There were no significant differences between the FMT and MET group at day 0, but both groups were significantly different from HC. After FMT, microbial communities clustered closer to healthy dogs at day 7 and day 28.

In contrast, administration of MET showed a larger difference in microbial communities compared to healthy dogs at day 7 (based on an increased effect size R=0.946), and microbial communities had still a large size effect compared to HC at day 28.
